# Anti-Chaff Jamming Method of Radar Based on Real Dataset and Residual Attention Model

**DOI:** 10.3390/s25092663

**Published:** 2025-04-23

**Authors:** Shuolei Li, Bin Liu, Lin Zhou, Jingping Liu

**Affiliations:** School of Electronic and Optical Engineering, Nanjing University of Science and Technology, Nanjing 210094, China; lishuolei@njust.edu.cn (S.L.);

**Keywords:** chaff jamming, dataset, radar, HRRP, residual, attention mechanism

## Abstract

As a typical and widely used passive jamming method, chaff clouds have a strong interference effect on radar that remains a significant challenge effectively to counteract. It is exceedingly necessary to improve the anti-chaff jamming ability of radars. In this paper, we address this challenge by proposing an effective residual attention network named RA-Net. Specifically, we introduce an attention mechanism that enables the network to focus on the most informative and stable hierarchical features of the high-resolution range profile (HRRP) data, significantly improving the model’s feature extraction capability and overall performance. In addition, we address the limitation of insufficient measured chaff cloud echo data by establishing a remarkably rich and diverse data set of chaff cloud HRRP data through extensive field experiments. This dataset serves as a valuable resource and a critical foundation for advancing HRRP recognition research in this domain. Experimental results on measured HRRP data demonstrate that RA-Net achieves superior recognition accuracy of 97.10%, outperforming traditional methods, and also exhibits outstanding generalization capability. These results establish RA-Net as a new benchmark for chaff cloud HRRP recognition.

## 1. Introduction

Chaff jamming has long been a key factor impacting the normal operation of radar systems. Due to its simple production, low cost, and effective jamming capabilities, chaff has been widely used in military conflicts and has become a major research focus in the fields of jamming and anti-jamming technology [1,2,3,4,5,6]. Chaff jamming significantly degrades radar detection performance, often leading to false target detection. Therefore, it is of great significance to carry out research on anti-chaff jamming of radar.

In order to improve the anti-chaff jamming ability of radars, [7] investigated target recognition technology using pulse Doppler radar based on bandwidth characteristics. In [8], the authors simulated and analyzed target echo signals under chaff cloud jamming and assessed the impact of the chaff cloud jamming effect on radio fuzes. In [9], the authors conducted research based on the sparseness characteristics of the chaff cloud echo signal and target echo signal. In [10], two alternative methods were proposed to estimate the direction of arrival (DOA) of the target in the presence of chaff jamming. Omnidirectional radar cross-section (RCS) simulations of chaff clouds and ships in CST electromagnetic simulation software have been carried out as well. For instance, the high-resolution range profile (HRRP) and polarization characteristics of chaff clouds and ships were analyzed in [11]. In [12], the authors proposed an anti-chaff jamming method based on oblique projection polarization filtering, then simulated and analyzed the validity of the proposed method to expand the application scope of polarization filtering. In [13], the authors first briefly described the development status of chaff jamming, then summarized the research status of anti-chaff jamming methods based on spectral spreading, polarization characteristics, Doppler difference, echo signal characteristics, machine learning, and more. However, due to the lack of real experimental data on chaff clouds, the methods mentioned above all based their analysis on simulated data, meaning that practical performance still needs to be further verified. For this reason, it is exceedingly necessary to establish a real chaff cloud dataset.

Several researchers have explored anti-chaff methods based on two-dimensional range–Doppler profiles and validate their effectiveness [14]; however, these methods require additional computational resources. Other researchers [15,16,17] have focused on polarization information; however, methods based on polarization information typically involve more complex hardware and are complicated to implement. In [18], the authors investigated the HRRP characteristics of chaff clouds and provided theoretical support for the development of jamming and anti-jamming capabilities in radio fuzes. In [19,20], the authors proposed chaff identification methods based on the range–Doppler features of radars. In [21], the authors utilized range profile data as input to a convolutional neural network (CNN) in order to discriminate chaff clouds from ships. However, the generalization performance of the network was not validated.

In this paper, we propose an effective recognition framework named RA-Net that is specifically designed to enhance the accuracy and robustness of classification. A key component of RA-Net is its integration of an attention mechanism. This enables the network to focus on the most informative and stable hierarchical features, effectively improving its feature extraction capability as well as the overall performance of the model. Furthermore, we construct a comprehensive and diverse chaff cloud HRRP dataset through extensive field experiments, providing a solid foundation for further research on HRRP recognition of chaff clouds in complex real-world scenarios. Experimental results on this HRRP dataset reveal the superiority of our method over traditional approaches, achieving outstanding recognition accuracy of 97.10% along with excellent generalization performance.

## 2. Chaff Cloud HRRP Dataset

**Figure 1:**
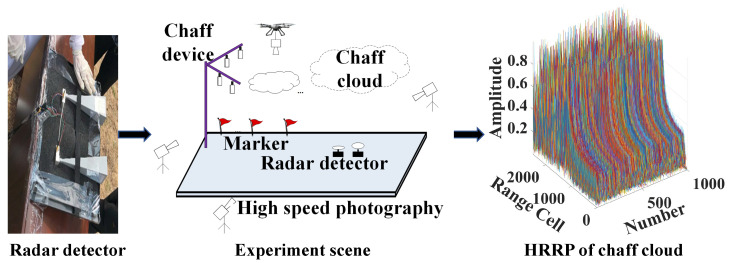
The program of the field experiment for chaff cloud HRRP data acquisition.

In order to establish a comprehensive chaff cloud HRRP dataset, we conducted a large number of field experiments. A frequency modulation continuous wave radar was used to collect chaff cloud echo signals. The modulation signals consisted of a triangular wave, the modulation voltage range was 6–9 v, the modulation period was 0.5 ms, the frequency range of the radio frequency (RF) signal was 34 to 35.5 GHz, the bandwidth of the RF signal was 1.5 GHz, and the range resolution was 10 cm. The aim of the field experiment was to carry out extensive chaff cloud diffusion experiments under different conditions. The acquisition time for the chaff cloud echo signal in each field experiment was set to the range of 3–6 s, while the sampling frequency was 2 MHz. The field experiment program for chaff cloud HRRP data acquisition is shown in Figure 1.

HRRP can be considered to be the amplitude of the coherent summations of the complex time returns from target scatterers in each range cell, which represents the reflected radar intensity versus the range along the target extent [22]. Because our radar operates in the millimeter-wave frequency band and the size of the chaff cloud is much larger than the wavelength of the radar when the chaff cloud is in the diffusion stage, the radar can effectively divide the chaff cloud into many range cells. The radar signals from scattering centers within the same range cell are coherently summed into a single signal for that range cell. By applying the fast Fourier transform of the chaff cloud echo signal for each period, we can obtain the HRRP data of the chaff cloud.

**Figure 2:**
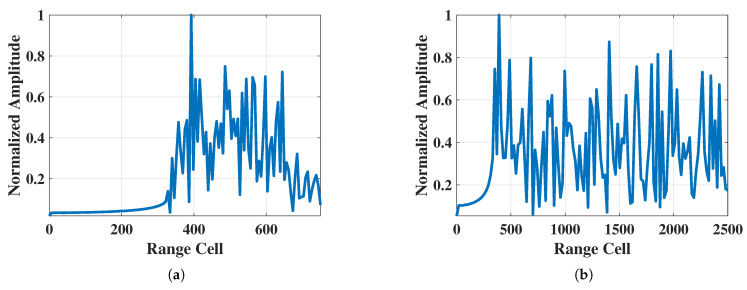
Measured chaff cloud HRRP sample data from the standard chaff cloud HRRP dataset and the non-standard chaff cloud HRRP dataset: (**a**) standard chaff cloud HRRP data and (**b**) non-standard chaff cloud HRRP data.

Two measured chaff cloud HRRP dataset versions are provided: in the standard chaff cloud HRRP dataset, the chaff length covers 4 mm and 15 mm and the interference band is the X band and the Ka band, while in the non-standard chaff cloud HRRP dataset the chaff length covers 4.1 mm, 8.2 mm, 9.6 mm, 12 mm, 14.5 mm, 17.7 mm, and 20 mm and the interference band is from the C band to the Ka band. The measured chaff cloud HRRP sample data from the standard chaff cloud HRRP dataset and the non-standard chaff cloud HRRP dataset are shown in Figure 2.

## 3. Proposed Methods

In this section, we show the details of the proposed RA-Net, including the overview of the network architecture, the fundamental components of the network, and the loss function.

### 3.1. Design of RA-Net

**Figure 3:**
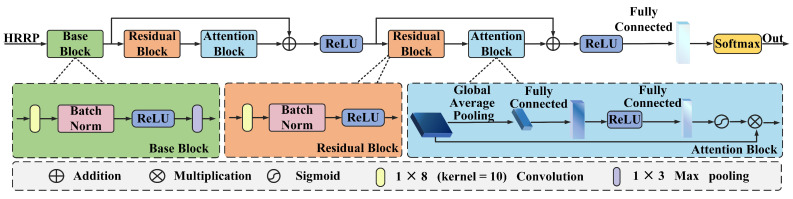
Architecture of the proposed method.

The RA-Net architecture employs a modular design combining a base block, residual block, attention block, and fully connected layer, followed by a softmax layer. The network takes the HRRP data as input and outputs the final predictions. The overall architecture of RA-Net is shown in Figure 3. The base block is used to extract initial low-level features from the input HRRP data, then the output of the base block is passed to the residual block to enhance gradient flow and enable deeper feature extraction without the risk of degradation. The attention block improves the model’s ability to focus on relevant features for enhanced recognition accuracy.

### 3.2. Residual Block

As the network depth increases, the accuracy of the network becomes saturated; for this reason, even networks with great depth face the problem of performance degradation. To ensure maximum information flow between the layers of the network, we link all of the matched feature maps by introducing residual blocks, then reuse the low-level features in the higher levels for improved classification accuracy.

Each residual block in our proposed network is composed of a convolutional layer, batch normalization layer, and ReLU layer. In the convolutional layer, the convolutional kernel is particularly effective for extracting local features along one dimension, making it well suited for processing HRRP data. The output of the convolutional layer is normalized in the batch normalization layer. Batch normalization helps to stabilize and accelerate training by normalizing the feature distributions to have zero mean and unit variance, which reduces internal covariate shift. The normalized feature map is then passed through the ReLU activation function. The ReLU layer introduces nonlinearity and sparsity, which is essential for learning complex data representations and also helps to improve computational efficiency and reduce the risk of overfitting. Mathematically, the output of the residual block can be expressed as

(1) $$y=F\left(x,w_{c},w_{b}\right)+x,$$

where *x* is the input, the function *F* represents residual mapping to be learned by the convolutional layer, batch normalization layer, and ReLU layer, $w_{c}$ and $w_{b}$ represent the weights of the convolutional layer and batch normalization layer, respectively, and *y* is the final output.

### 3.3. Attention Block

Considering that not all features in HRRP data are equally important, certain regions may contain more discriminative information. Thus, we add an attention block, enabling the network to assign higher weights to the important features while suppressing irrelevant features and noise. Focusing on informative information improves the network’s efficiency and accuracy during chaff cloud jamming identification.

First, the input feature map is processed through a global average pooling layer. Then, the global average pooling layer computes the spatial average of the feature map with (2), effectively summarizing the global spatial information into a single scalar. This operation compresses the feature map from the H×W spatial dimension to a 1×1 vector:

(2) $$g_{\mathrm{pool}\;}=G_{pool}\left(X\right)=\frac{1}{H\times W}\sum_{i=1}^{H}\sum_{j=1}^{W}x_{ij}$$

where $G_{pool}\left(X\right)$ represents the global average pool, *X* is the input feature map, and $x_{ij}$ denotes the value of input feature map at position (i,j).

The compressed global feature vector is passed through two fully connected layers connected by the ReLU layer. The output of the second fully connected layer is processed through a sigmoid activation function to scale the recalibrated weights to range [0,1]. The scaled weights represent the relative importance of the HRRP data, which can be expressed as follows:

(3) $$s=\sigma \left(w_{2}\delta \left(w_{1}g_{\mathrm{pool}\;}+b_{1}\right)+b_{2}\right)$$

where $g_{\mathrm{pool}}$ is the output of the global average pooling layer, $w_{1}$ and $w_{2}$ are the learnable weight matrices of the fully connected layers, $b_{1}$ and $b_{2}$ are the learnable biases of the fully connected layers, and δ and σ refer to the ReLU function and sigmoid activation function, respectively.

The original feature map is multiplied by the learned attention weight. This operation reweights the features according to their importance as determined by the attention mechanism:

(4) $$F_{\mathrm{out}\;}=F_{\mathrm{in}\;}⊗s$$

where $F_{\mathrm{in}\;}$ is the input feature map, $F_{\mathrm{out}\;}$ is the recalibrated output, *s* represents the attention weight, and ⊗ denotes element-wise multiplication.

### 3.4. Loss Function

In order to train our proposed network to achieve greater performance, we choose the cross-entropy loss as our optimization function. The cross-entropy loss formula is defined as follows:

(5) $$\;\mathrm{loss}\;=-\frac{1}{N}\sum_{n=1}^{N}\sum_{i=1}^{K}t_{ni}\ln y_{ni}$$

where *N* is the number of HRRP samples, *K* is the number of classes, $t_{ni}$ indicates that HRRP sample *n* belongs to class *i*, and $y_{ni}$ is the output for HRRP sample *n* for class *i*, which in this case is the value from the softmax function; in other words, $y_{ni}$ is the probability of the network associating observation n with class *i*.

## 4. Experiments

In this section, we use the combined measured chaff cloud HRRP dataset to conduct extensive experiments evaluating the proposed RA-Net against other methods in terms of recognition performance and generalization performance.

### 4.1. Training and Test Sets

The experiments involved two datasets, namely, the chaff cloud HRRP dataset and the target HRRP dataset. The chaff cloud HRRP dataset described in Section 2 consists of 12,000 data samples, while the target HRRP dataset is derived from corner reflector echo signals at varying distances, and also contains 12,000 data samples. The two datasets were both divided into training and test sets at a training:test ratio of 7:3. The training set included 8400 chaff cloud HRRP samples and 8400 target HRRP samples, while the test set comprised 3600 chaff cloud HRRP samples and 3600 target HRRP samples. This experimental setup ensures a rigorous evaluation of radar performance under controlled conditions.

### 4.2. Training Details

RA-Net was trained in MATLAB R2024b, a computational platform developed by MathWorks. This version incorporates the Deep Learning Toolbox, Parallel Computing Toolbox, and GPU-accelerated optimization support, enabling efficient execution of large-scale data training and complex model optimization tasks. The platform was operated on hardware equipped with a high-performance multicore 12th-Gen Intel Core i9-12900K CPU and NVIDIA GeForce RTX 3080 GPU. The platform’s interactive debugging interface and real-time visualization tools significantly enhanced the efficiency of model training and experimental iteration.

**Figure 4:**
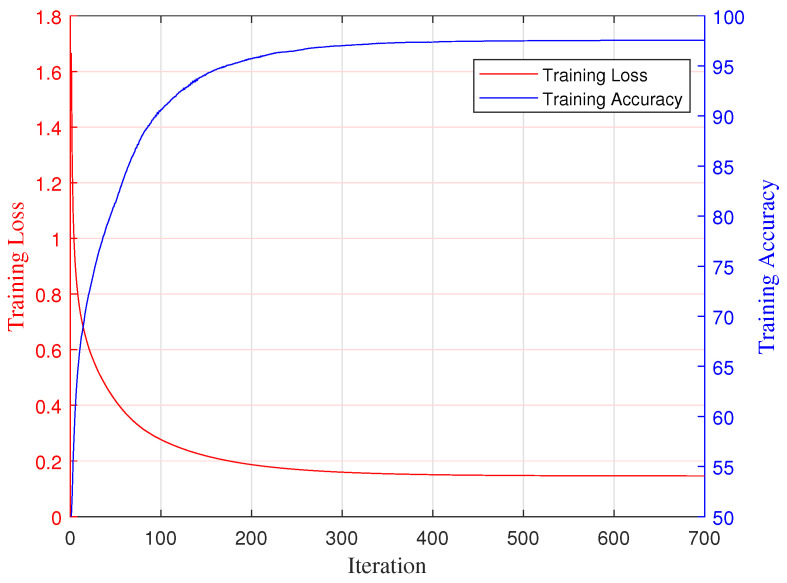
Training progress of our proposed RA-Net.

We trained our RA-Net for 700 epochs on all training sets using a single NVIDIA GeForce RTX 3080 GPU. The batch size used to evaluate the gradient of the loss function and update the weights was 10. The initial learning rate was set at 0.001, and the learning rate schedule reduced the learning rate by a factor of ten at every 10 epochs. The root mean square propagation optimizer (rmsprop) was used to train the proposed neural network. The training progress of RA-Net is shown in Figure 4.

### 4.3. Recognition Performance

**Figure 5:**
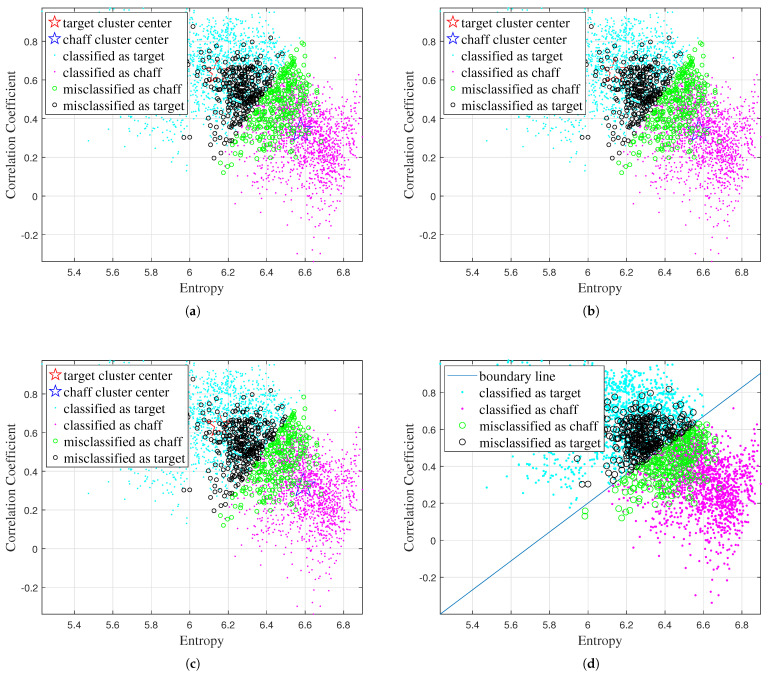
Classification results obtained by different traditional algorithms using the entropy and correlation coefficient features: (**a**) K-Means, (**b**) Fuzzy C-Means, (**c**) FCM-σ, (**d**) SVM.

**Figure 6:**
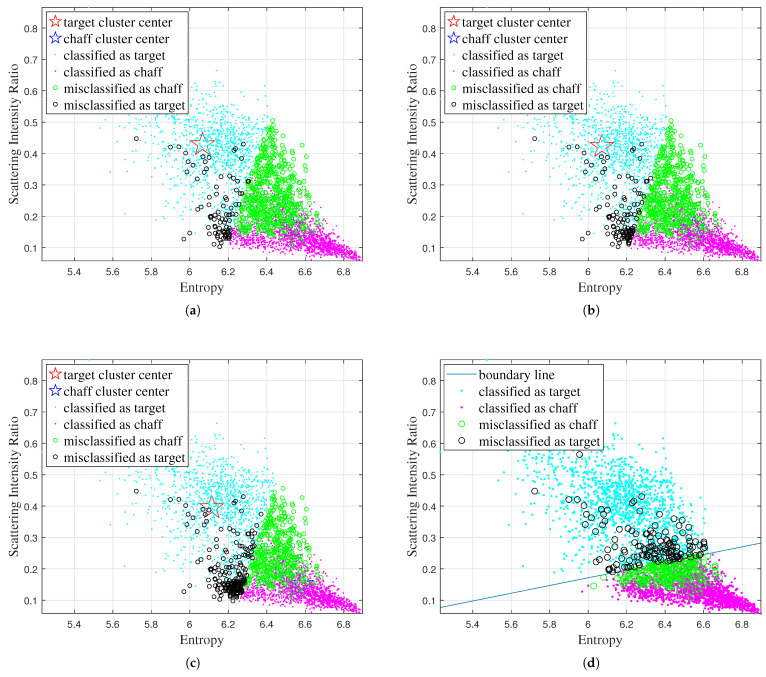
Classification results obtained by different traditional algorithms using the entropy and scattering intensity ratio features: (**a**) K-Means, (**b**) Fuzzy C-Means, (**c**) FCM-σ, (**d**) SVM.

**Figure 7:**
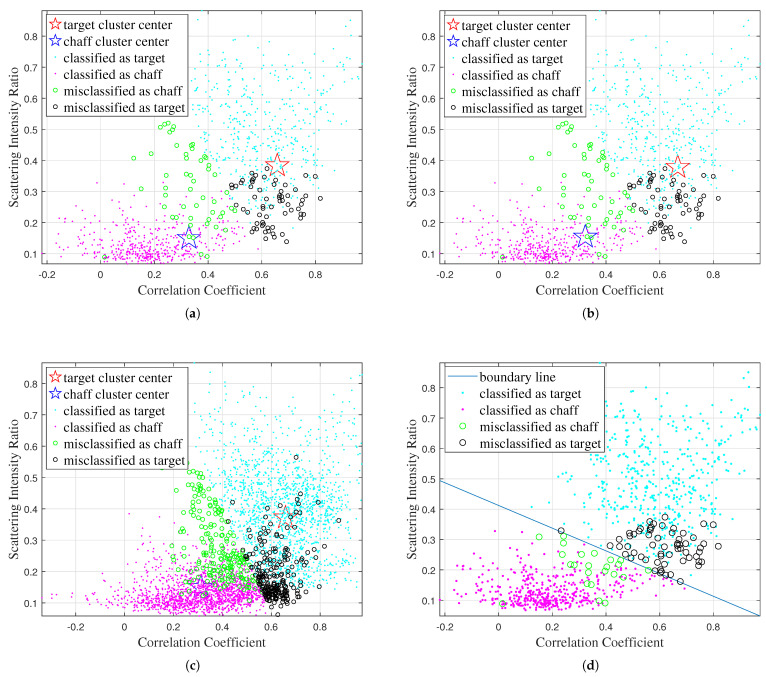
Classification results obtained by different traditional algorithms using the correlation coefficient and scattering intensity ratio features: (**a**) K-Means, (**b**) Fuzzy C-Means, (**c**) FCM-σ, (**d**) SVM.

**Figure 8:**
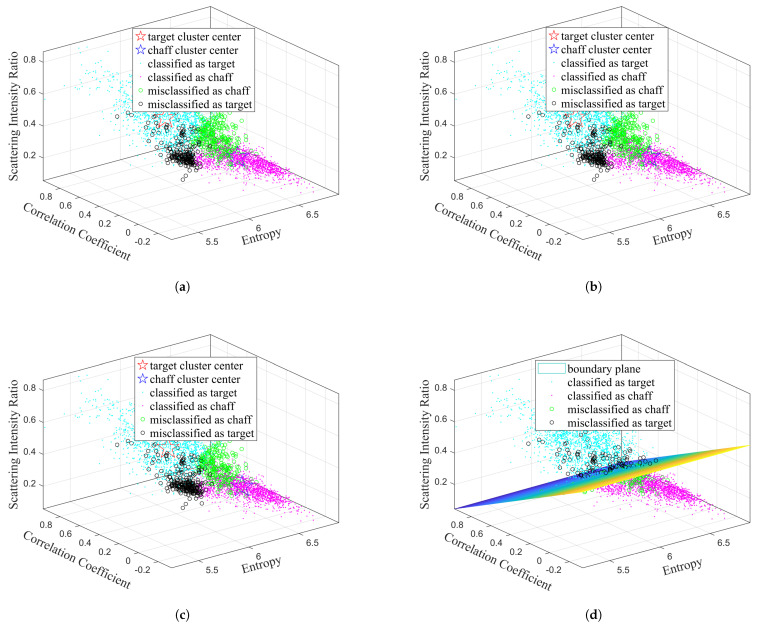
Classification results obtained by different traditional algorithms using the entropy, correlation coefficient, and scattering intensity ratio features: (**a**) K-Means, (**b**) Fuzzy C-Means, (**c**) FCM-σ, (**d**) SVM.

Firstly, the classification results obtained by the different traditional algorithms using various combinations of features are shown in Figure 5, Figure 6, Figure 7, and Figure 8, respectively, while the recognition performance results are shown in Table 1. The definitions for the entropy, correlation coefficient, and scattering intensity ratio are shown in Appendix A.

As shown in Table 1, the traditional machine learning algorithms exhibit limited discriminative capability due to their strong dependence on features, and have different recognition performances with different feature inputs. The K-Means, Fuzzy C-Means, FCM-σ, and SVM models achieve the best recognition performance when using correlation coefficient and scattering intensity ratio as input.

In Table 2, we present a comparative analysis of classification performances on the test set achieved by the proposed model and several traditional methods. The results clearly demonstrate the superiority of the proposed model in achieving high classification performance compared to several traditional clustering methods: K-Means, Fuzzy C-Means and FCM-σ, support vector machine (SVM), Inception-v1, and the baseline CNN deep learning method. The baseline CNN architecture is identical to that of RA-Net except that it does not include the residual blocks or attention blocks, and the parameter settings of the other parts are also identical.

As shown in Table 2, the CNN method demonstrates a clear improvement over SVM, achieving 94.35% recognition performance. This result emphasizes the ability of deep learning to automatically learn and extract discriminative features, outperforming traditional machine learning techniques that rely on manually extracted features. Inception-v1 outperforms CNN, achieving 95.17% accuracy due to its ability to efficiently extract multiscale features, reduce computational cost, and improve training stability. Multiscale feature extraction enables Inception-v1 to extract both fine and coarse details from the input, resulting in enhanced recognition performance, while its use of 1 × 1 convolutions for dimensionality reduction reduces computational cost while maintaining essential feature representations.

The proposed model achieves the highest recognition performance of 97.10%, representing a 1.93% absolute improvement over Inception-v1, a 2.75% absolute improvement over CNN, and an even larger margin over SVM and clustering-based methods. This significant performance boost and substantial improvement can be attributed to the inclusion of architectural innovations via the residual blocks, which facilitate the propagation of features and gradients across network layers, as well as the incorporation of an attention mechanism, which dynamically recalibrates feature importance, allowing the model to focus on the most discriminative information. These architectural enhancements allow the proposed model to set a new benchmark for classification tasks, offering a robust and highly effective solution for scenarios requiring accurate recognition.

### 4.4. Generalization Performance

In aforementioned experiments, the test set was designed to cover as many chaff cloud diffusion scenarios as possible. However, in some circumstances (for example, during battle), the diffusion patterns of the chaff cloud can vary significantly, and may differ from those in the test set. In such cases, the generalization performance of RA-Net becomes an important criterion for evaluation.

In order to obtain data distinct from the training and test sets in previous experiments, we conducted new chaff cloud diffusion experiments under varying conditions, including different heights above the ground, different horizontal distances from the radar, and different wind speeds. As a result, we collected 1000 new chaff cloud HRRP samples. In addition, we acquired 1000 new target HRRP samples by varying the height above the ground and horizontal distance from the radar. We refer to this newly collected dataset of 2000 unused samples as NUDS (New Unused Data Set).

Table 3 depicts the classification performance of the proposed model compared with several traditional methods on the NUDS dataset. It can be seen that the proposed RA-Net still achieves the highest recognition performance of 94.85%, demonstrating its robustness and adaptability when handling complex HRRP data under varying conditions.

These results emphasize the importance of our model architecture design and highlight the limitations of traditional methods, showing that the proposed method is well suited for real-world applications that require reliable classification across diverse datasets.

## 5. Conclusions

In this paper, we have addressed the critical challenge of chaff cloud HRRP recognition in complex electromagnetic environments by proposing a novel and effective method called RA-Net. To overcome the limitation of insufficient measured chaff cloud echo data, we constructed a rich and diverse chaff cloud HRRP dataset through extensive field experiments. This dataset not only supports our experiments but also serves as a benchmark for advancing research on radar anti-chaff jamming. Furthermore, we integrated an attention mechanism into the proposed network to extract more informative and stable hierarchical features from HRRP data and improve network performance. Experimental results on the measured HRRP data reveal that our proposed method achieves recognition accuracy of 97.10% and exhibits exceptional generalization performance compared to other methods. These advancements suggest that RA-Net effectively bridges the gap between laboratory-based models and real-world deployment requirements. Future work will extend this framework and explore its broader applicability in anti-chaff jamming techniques.

## Figures and Tables

**Table 1:** Recognition rates of different traditional algorithms across various feature combinations. Here, A, B, and C represent the entropy, correlation coefficient, and scattering intensity ratio, respectively.

Features	K-Means	Fuzzy C-Means	FCM-σ	SVM
A + B	83.17%	83.20%	83.20%	84.33%
A + C	81.74%	81.90%	82.59%	89.53%
B + C	88.71%	88.51%	88.02%	93.25%
A + B + C	86.56%	86.53%	86.61%	93.20%

**Table 2:** Classification performance of the proposed model compared with several traditional methods on the test set. The best result is highlighted in bold.

Methods	Recognition Performance
K-Means	88.71%
Fuzzy C-Means	88.51%
FCM-σ	88.02%
SVM	93.25%
Inception-v1	95.17%
CNN	94.35%
**RA-Net**	**97.10%**

**Table 3:** Classification performance of the proposed model compared with several traditional methods on the NUDS dataset. The best result is highlighted in bold.

Methods	Recognition Performance
K-Means	88.30%
Fuzzy C-Means	88.10%
FCM-σ	88.50%
SVM	91.50%
Inception-v1	94.50%
CNN	90.05%
**RA-Net**	**94.85%**

## Data Availability

The original contributions presented in this study are included in this paper. For more detailed information, please contact the corresponding author directly.

## Appendix A

### Appendix A.1. Entropy

The concept of entropy is introduced to assess the complexity of the HRRP sequences of the chaff cloud and target. Let the HRRP sequences be represented as {S(l),l=1,2,…,N}. We first normalize the HRRP sequences using the following formulas:

(A1) $$\begin{array}{cc} \bar{S(l)} & =\frac{S(l)}{S^{\prime }}, \end{array}$$

(A2) $$\begin{array}{cc} S^{\prime } & =\sum_{l=1}^{N}S\left(l\right). \end{array}$$

The waveform entropy of the HRRP sequences S(l) can be expressed as follows:

(A3) $$E\left(S\right)=-\sum_{l=1}^{N}\bar{S(l)}\log_{2}\bar{S(l)}.$$

The waveform entropy, denoted as E(S), quantifies the inherent dispersion characteristics in the distribution of HRRP sequences. A lower entropy value corresponds to a more concentrated energy distribution within the target’s scattering centers. In contrast, elevated entropy values demonstrate increased dispersion in the energy distribution of the scattering centers.

### Appendix A.2. Correlation Coefficient

The geometric differences between the target and the chaff cloud lead to variations in the distribution of the scattering points. In turn, these differences affect the characterization of the HRRP sequences and influence the correlation of the HRRP sequences.

The correlation coefficient of the HRRP sequences between different echo periods is defined as follows:

(A4) $$\rho \left(r,k\right)=\frac{\mathrm{cov}\left[\left(S^{r}-\bar{S^{r}}\right),\left(S^{k}-\bar{S^{k}}\right)\right]}{\sigma \left(S^{r}-\bar{S^{r}}\right)\sigma \left(S^{k}-\bar{S^{k}}\right)}$$

where cov represents the covariance operation, σ represents the standard deviation operation, $S^{r}$ and $S^{k}$ represent the HRRP sequences of different echo periods, and $\bar{S^{r}}$ and $\bar{S^{k}}$ denote the expectation of the HRRP sequences for different echo periods.

The average correlation coefficient of the HRRP sequences across *N* different echo periods is defined as follows:

(A5) $$c=\sum_{i=2}^{N}w_{i}\rho \left(1,i\right),$$

(A6) $$w_{i}=\frac{2(N+1-i)}{N(N-1)}.$$

### Appendix A.3. Scattering Intensity Ratio

First, the HRRP sequences for each period are sorted in descending order according to the amplitude. The sorted HRRP sequences of each period can be expressed as $\left\{x_{1}\left(1\right),x_{1}\left(2\right),\ldots ,x_{1}\left(n\right)\right\}$.

To calculate the scattering intensity ratio, we determine the power values of the first m strong scattering points in the sorted HRRP sequences and compare them to the total power value of the entire HRRP sequence. The scattering intensity ratio can then be expressed as

(A7) $$K_{m}=\frac{\sum_{i=1}^{m}x_{1}^{2}\left(i\right)}{\sum_{i=1}^{n}x_{1}^{2}\left(i\right)}.$$
